# Acute Toxicity and Tissue Distribution of Cerium Oxide Nanoparticles by a Single Oral Administration in Rats

**DOI:** 10.5487/TR.2009.25.2.079

**Published:** 2009-06-01

**Authors:** Eun-Jung Park, Young-Kwon Park, Kwangsik Park

**Affiliations:** 13 College of Pharmacy, Dongduk Women’s University, 23-1, Wolgok-dong, Seongbuk-gu, Seoul, 136-714 Korea; 23 Faculty of Environmental Engineering, College of Urban Science, University of Seoul, 90 Jeonnong-dong, Dongdaemun-gu, Seoul, 130-743 Korea

**Keywords:** Cerium oxide nanoparticles, Acute toxicity, Tissue distribution, Rats

## Abstract

Cerium oxide nanoparticles (size: 30 nm) were prepared by the supercritical synthesis method, Acute oral toxicity and tissue distribution of the nanoparticles were evaluated by a single administration in rats. Oral administration of the nanoparticles to the rats did not lead to death when the animals were treated by a dose of 5 g/kg (high dose) as well as 100 mg/kg (low dose). Abnormal clinical signs, changes in serum biochemistry and hematology were not observed in high-dose treated group compared to the vehicle control group. Lesions in liver, lung and kidney were not observed in high-dose treated group by histopathological examination. Tissue distribution analysis in liver, kidney, spleen, lung, testis and brain was performed on day 1, day 7 and day 14 after treatment. The average values of the accumulated cerium oxide nanoparticles were elevated in all tissues but statistical significance was only shown in lung. Low levels of tissue distributions after a single oral administration seem to be the low bioavailability of the nanoparticles.

## INTRODUCTION

Recently, the potentially hazardous health effects of nanoparrticles are emerging issues and publications on the toxicities of nanoparticles are rapidly increasing (Kipen *et al*., 2005; Kagan *et al*., 2005; Oberdorster *et al*., 2005; Curtis *et al*., 2006; Hardman, 2006; Priestly *et al*., 2007). Various industrial nanomaterials are made from metal or carbon. Metal nanoparticles such as titanium oxide, cerium oxide, silver, gold, and alumina have been widely used for industrial purposes. Among them, cerium oxide nanoparticles (CNP) may be the most important nanomaterials with wide ranges of applications for solar cells, fuel cells, gas sensors, oxygen pumps, and glass/ceramic applications (Gao *et al*., 2006). As the applications of CNP increase, the concerns about their potential human toxicity effects and their environmental impact were also increased. However, toxicity data for CNP relating to human health are limited and only a few results from basic studies have been published. Furthermore, the toxicity of CNP is still controversial, especially regarding the oxidant/antioxidant effect (Thill *et al*., 2006: Lin *et al*., 2006: Schubert *et al*., 2006, Park *et al*., 2008a).

Regarding the induction of reactive oxygen species (ROS) by CNP, clear explanation of the mechanisms has never been tried. The potential redox properties of CNP seem to play a key role in the generation of ROS. Lin *et al*. (2006), proposed that $Ce^{3+}$ produced by the reduction of $Ce^{4+}$ interacts with oxygen molecules ($O_2$), and generates superoxide anions. The two superoxide anion molecules interact and are converted to hydrogen peroxide, and then the hydrogen peroxide converts to hydroxyl radicals. However, totally different chemical reactions for CNP were suggested by Das et al that the presence of the mixed valence states of $Ce^{3+}$ and $Ce^{4+}$ on the surface of the nano-ceria act as an anti-oxidant that allows the nanoparticles to scavenge free radicals from the culture system (Das *et al*., 2007). $Ce^{3+}$ reacts with hydroxyl radicals ($Ce_2O_3 + 2[\cdot OH] \rightarrow 2CeO_2 + H_2O$). They proposed this radical scavenging of CNP is the mechanism of neuroprotection in the spinal cord neurons of adult rats. Schubert *et al*. also suggested the CNP is protective in cultured HT22 cells, which are derived from the rodent nervous system (Schubert *et al.*, 2006). This suggests that some discrepancies on the toxicity of CNP have been existed. Furthermore, the toxicity exerted by CNP in vivo and in vitro may be different.

In our previous study, we evaluated the cytotoxic effect of CNP using BEAS-2B cells which are derived from human bronchial epithelia normal cells (Park *et al.*, 2008a). CNP induced ROS generation and decreased the intracellular glutathione (GSH) levels, finally caused apoptotic cell death determined by caspase-3 activation and chromosome condensation. Based on the results of *in vitro* toxicity tests, we tired to investigate the *in vivo* toxicity of CNP in rats and to evaluate the target organ of CNP when administered orally.

## MATERIALS AND METHODS

***Preparation of cerium oxide nanoparticles (CNP).*** The cerium oxide was prepared by the supercritical synthesis method published in previous report (Park *et al.*, 2008a). The synthesis process is outlined as follows. Distilled water, pumped into the system using a high pressure pump, was heated to a moderate temperature by a heat exchanger and heated further to a desired temperature by a pre-heater. The solution of the metal precursor $Ce(NO_3)_3$ and ammonia water for pH control, was pumped into the system using high pressure pumps, and mixed with supercritical water at the mixing point located above the reactor. A sufficient reaction time for the formation of CNP was provided by the reactor in order to maintain a supercritical condition. The synthesized CNP was collected by filter, and this was followed by spray drying to obtain the final product. The crystal structure of CNP was confirmed by powder X-ray diffraction (XRD) patterns using monochromic Cu-Ka radiation (Rigaku, D/MAX III). The average crystallite size was also measured by the X-ray line broadening technique employing the Scherrer formula using the profiles of the (1 1 1) peak. SEM (Scanning Electron Microscope) images of cerium oxide nanoparticles were obtained by a Phillips SEM-535M microscope operating at an acceleration voltage of 20 kV. The samples were prepared by sprinkling the powder oxides onto double-sided sticky tape and mounting them on a microscope stub. The samples were coated with sputtered gold. Fig. 1 shows the morphology and crystallite size of cerium oxide (30 nm-size). Test for acute oral toxicity was performed according to the Test Guidelines issued by Korea Food and Drug Administration. The high dose (5 g/kg) and low dose (100 mg/kg) were based on the preliminary lethality test.

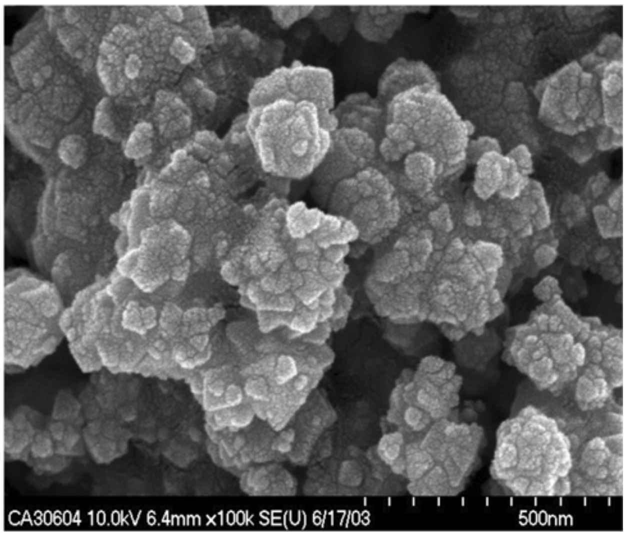

**Fig. 1.** Image of cerium oxide nanoparticles shown by a scanning electron microscope.

***Animals and treatment.*** Male SD rats were purchased from Orient-Bio Animal Company (Seongnam, Gyunggi-do, Korea) and were allowed to adapt to the animal room conditions prior to initiation of the study. The environmental conditions were a temperature of 23 ± 1°C; relative humidity of 55 ± 5%; and a 12 h light/dark cycle. CNP (30 nm size) was suspended in distilled water, and sonicated for 30 min before treatment. CNP was delivered orally by a single dose of 100 mg/kg-day (Low dose) and 5 g/kg-day (High dose). The number of animals is 4 in control group and 6 in treated group. Then, the animals were sacrificed on day 1, 7, and 14 after treatment.

***Hematological and serum biochemical assays.*** Blood samples were collected on day 1 after treatment for hematological examination and serum biochemical analysis. Hematological items such as blood cell counting were measured using a hematological auto-analyzer (HemaVet850, CDC Tech.Co., U.S.A.). For serum biochemical examination, blood sample were allowed to clot and centrifuged to separate serum. The sera were stored in the -80°C freezer prior to analysis of biochemical items such as total protein and albumin by using an auto-analyzer (Hitachi7180, Hitach, Japan).

***Histopathology.*** All organs were fixed in 10% (v/v) neutral buffered formalin and processed using routine histological techniques. After paraffin embedding, 3 μm sections were cut and stained with hematoxylin and eosin (H&E) for histopathological evaluations.

***CNP analysis in tissues.*** Tissues of liver, kidney, spleen, lung, testis and brain were collected from rats of control group and high-dose treated group on day 1, 7, and 14 after treatment. The wet tissues were dehydrated using freeze-dryer (Ilshin Co, Yangju, Korea) and the freeze-dried tissues were digested in the solution of $HNO_3$ and $H_2O_2$ using microwave digestion system (Milestone, Sorisole, Italy). Tissue concentration of cerium was analyzed using ICP-MS (Elan6100/Perkin Elmer, USA) in the Korean Basic Science Institute (KBSI) in Seoul, Korea.

***Statistical analysis.*** Results of hematological assay, serum biochemical assay and tissue distribution analysis were presented as the mean ± standard deviation (S.D). The results of the chemically-treated groups were compared to those of the control group. The values were compared using the Student's *t*-test, and levels of significance were represented for each result ($p < 0.01$).

## RESULTS

***Mortality.*** After a single oral administration of CNP, animals were monitored once a day. Dead animals were not found in both low-dose treated group (n = 6) and high-dose treated group (n = 6) during the whole experimental period, 14 days after treatment. Significant differences in clinical signs such as general appearance and behavioral changes were not observed (Table 1).

**Table 1.** Mortality in rats after a single oral administration of cerium oxide nanoparaticles

| | Control | Low dose (100 mg/kg, po) | High dose (5 g/kg, po) |
|---|---|---|---|
| Day 1 | 0/4 | 0/6 | 0/6 |
| Day 7 | 0/4 | 0/6 | 0/6 |
| Day 14 | 0/4 | 0/6 | 0/6 |

Mortality is represented as the number of dead animals/total animals

***Hematological and serum biochemical assay.*** Table 2 shows the hematological parameters measured in this study. The values of white blood cell (WBC) count, red blood cell (RBC) count, hemoglobin (Hb), hematocrit (HCT), Mean corpuscular volume (MCV), mean corpuscular hemoglobin (MHC), mean corpuscular hemoglobin concentration (MCHC), and other parameter are listed. Statistically significant differences were not observed by a single dose of CNP (5 g/kg) ($p < 0.01$). List of serum biochemical parameters are also shown in Table 2. Serum concentration of total protein, albumin, creatinine, cholesterol, blood urea nitrogen (BUN), alanine aminotransferase (ALT), aspartate aminotransferase (AST), alkaline phosphatase (ALP), and $\gamma$-glutamyl transpeptidase ($\gamma$-GTP) are not statistically different between control group and CNP-treated group (5 g/kg, single dose) ($p < 0.01$).

***Histopathological findings.*** After a single oral administration of CNP (5 g/kg) to rats, the animals were sacrificed on day 14 and histopathological examination was performed in liver, lung and kidney. As shown in representative photo of H&E staining, no lesion was shown in liver, lung and kidney (Fig. 2). Inflammatory signs in the tissues from treated rats were not identified compared to the control group.

***Tissue distribution.*** CNP concentrations in liver, kidney, spleen, lung, testis, and brain were analyzed as the concentration of cerium in tissue by ICP-MS

**Table 2.** The values of hematological parameters and serum biochemistry in rats after a single dose of cerium oxide nanoparticles

| | Hematology | | | Serum biochemistry | |
|---|---|---|---|---|---|
| | Control | Treated | | Control | Treated |
| WBC (K/μl) | 6.6 ± 2.2 | 7.0 ± 2.5 | Total protein (g/dl) | 5.6 ± 0.2 | 5.5 ± 0.2 |
| RBC (M/μl) | 5.8 ± 0.2 | 6.0 ± 0.4 | Abumin (g/dl) | 2.5 ± 0.1 | 2.5 ± 0.1 |
| Hb (%) | 13.0 ± 0.1 | 12.7 ± 0.5 | Cholesterol (g/dl) | 92.0 ± 13.0 | 79.8 ± 99 |
| Hct (%) | 32.2 ± 1.1 | 34.9 ± 1.9 | Creatinin (mg/dl) | 0.2 ± 00 | 0.2 ± 00 |
| MCV (fl) | 55.1 ± 1.9 | 58.4 ± 1.6 | BUN (mg/dl) | 9.8 ± 0.5 | 10.2 ± 2.4 |
| MCH (pg) | 22.3 ± 0.6 | 21.4 ± 1.0 | AST (IU/l) | 104.3 ± 11.1 | 120.8 ± 36.8 |
| MCHC (g/dl) | 40.6 ± 0.9 | 36.5 ± 1.5 | ALT (IU/l) | 41.0 ± 7.1 | 41.6 ± 11.3 |
| Platelet (K/μl) | 969.0 ± 140.7 | 1035.2 ± 72.0 | r-GTP (IU/l) | 1.3 ± 2.5 | 0.2 ± 0.2 |
| Lymphocytes (%) | 66.2 ± 7.7 | 66.1 ± 2.6 | ALP (IU/l) | 1027.5 ± 172.2 | 950.8 ± 168.3 |
| Monocyte (%) | 4.8 ± 2.2 | 4.8 ± 1.1 | | | |
| Eosinophil (%) | 0.5 ± 0.2 | 0.5 ± 0.3 | | | |
| Basophil (%) | 0.2 ± 0.1 | 0.2 ± 0.1 | | | |

Each value represents mean ± S.D. (control; n = 4, treated; n = 6). Abbreviations are described in RESULTS

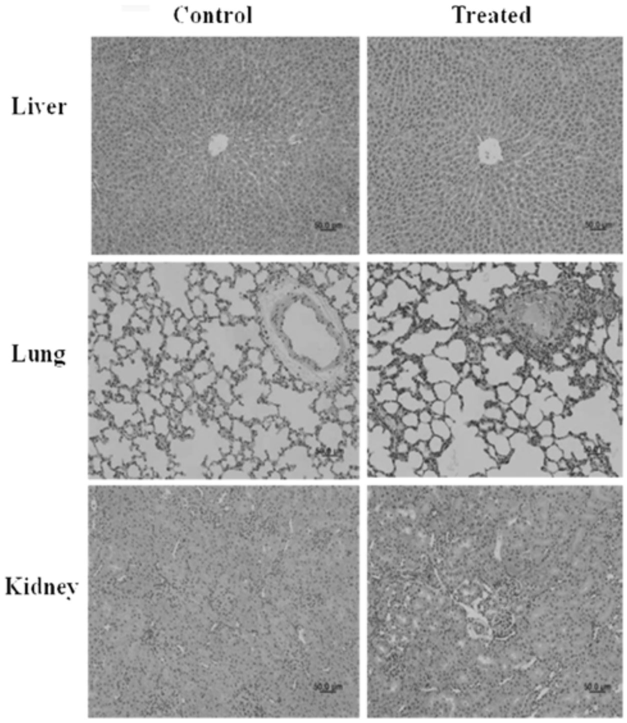

**Fig. 2.** Representative photomicrographs of liver, lung and kidney from control and treated rats. Rats were treated with a single oral dose of cerium oxide nanoparticles (5 g/kg) and sacrificed on day 14 after treatment.

(Elan6100/Perkin Elmer, USA) after a single oral administration of low dose (100 mg/kg) and high dose (5 g/kg), respectively. Animals were sacrificed on the designated days and tissues were collected for the chemical analysis. When rats were treated with CNP (100 mg/kg and 5 g/kg) and were sacrificed on day 1, accumulation of CNP in rat tissues seemed to be occurred in all tested tissues. However, the increase was not statistically significant except lung tissue (Fig. 3). In case of lung, it seemed to be main target organ of CNP by oral administration. When rats were treated with high dose, the level of ceria in lung was significantly increased on day 1 after treatment. The elevated level was decreased on day 7 and day 14 with time-dependent manner (Fig. 3A). In low-dose treated group, the ceria level in lung was also increased on day 1 with statistical significance and the increased level was decreased with time-dependent manner, which means the excretion of CNP (Fig. 3B).

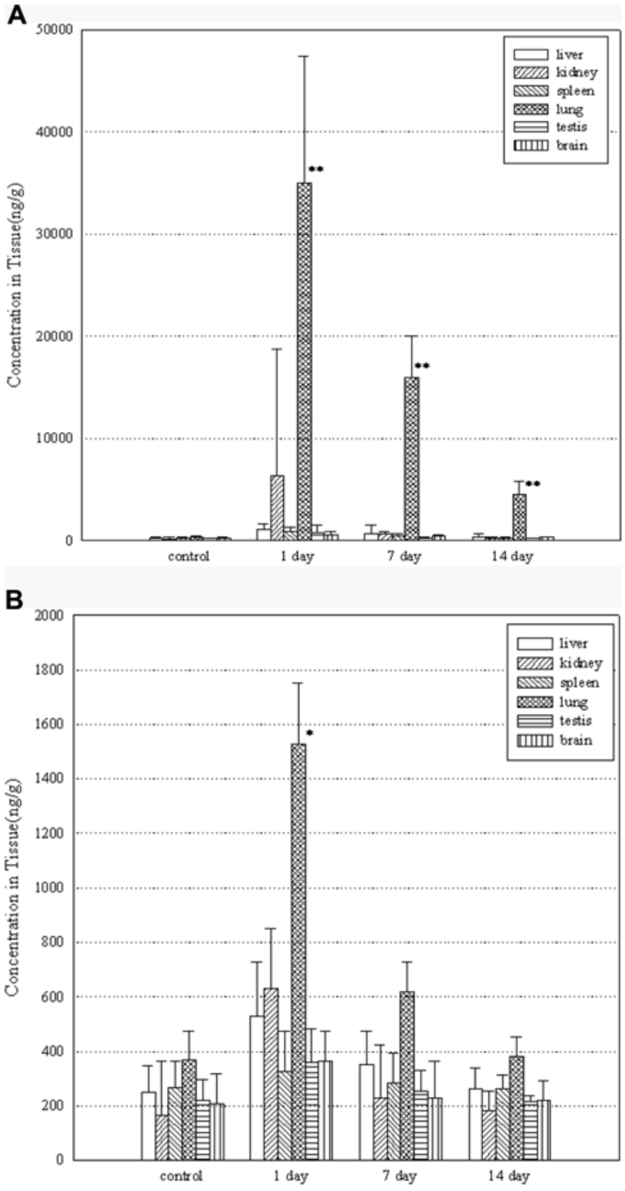

**Fig. 3.** Tissue distribution analysis in rats treated with a single oral dose of cerium oxide nanoparticle. Rats were treated with cerium oxide nanoparticles (100 mg/kg and 5 g/kg) and sacrificed on day 1, day 7, and day 14 (n = 6 on each sacrifice). Wet tissues of liver, kidney, spleen, lung, testis, and brain were digested in acidic solution and cerium concentration was analyzed using ICP-MS. **A**: 5 g/kg-treated, **B**: 100 mg/kg-treated. Statistically significant differences from the control group by the Student's *t*-test (**; $p < 0.01$, *; $p < 0.05$).

## DISCUSSIONS

As one of the toxic mechanisms of nanoparticles, the generation of ROS is being widely studied. There are some reports that ROS production and pro-inflammatory responses have been found in the cells treated with C60 fullerenes, Single Walled Carbon NanoTubes (SWCNTs), Multi Walled Carbon Nanoparticles (MWCNTs), silica nanoparticles, titanium oxide nanoparaticles, CNP and other metal particles (Sayes *et al.*, 2005; Hussain

*et al.*, 2005; Green *et al.*, 2005; Limbach *et al.*, 2007, Park *et al.*, 2008a, b; Park *et al.*, 2009a, b, c). It has been demonstrated that nanoparticles of various sizes and various chemical compositions attacked mitochondria, which are redox active organelles. Nanoparticles may alter ROS production and thereby may cause interference in the antioxidant defenses (Xia *et al.*, 2004; Foster *et al.*, 2006). However, the exact ROS generation mechanisms by nanoparticles are still unclear at this moment.

Previous studies suggested that CNP has different modes of action according to the types of cells. CNP did not show significant cytotoxicity in cultured T98G cells, derived from human brain cells or $H_9C_2$, derived from rat cardiomyocytes (Park *et al.*, 2008a) We do not know the exact mechanisms of cytotoxicities in different cells at this moment, but some cellular physiology related with antioxidant effects among the different types of cells, or some media environment may be one of the differences in exerting cytotoxicities. It seems that the oxidant/antioxidant effect of CNP should be more discussed and the preparation method of CNP may be another clue to this debating.

In this study, we investigated the single toxicity of CNP by oral administration using rats to elucidate the *in vivo* toxic mechanism of the nanoparticles. Because CNP caused oxidative stress and cellular apoptosis in cultured cells of *in vitro* study, we expected that CNP may also show strong toxicity *in vivo*. However, no single toxicity was shown both in low-dose treated group (100 mg/kg, a single oral administration) and in high-dose treated group (5 g/kg, a single oral administration).

No dead animals were found during the experimental period on day 14 after treatment (Table 1). Furthermore, serum biochemical parameters or hematological parameters were not changed with statistical significance (Table 2), which is an unexpected result. No acutetoxicity of CNP was observed in animal study, which showed cytotoxicity in BEAS-2B cells (10, 20, and 40 ppm for 4 days). It seemed that bioavailability of CNP was very low so that most of the administered CNP was not absorbed into blood stream but excreted with feces, which may be the reason of non-toxicity of CNP in *in vivo* study. After oral administration, the feces of the treated rats turned to be white which is the color of ceria pigment. Although we did not check the blood concentration level after administration at this time, the accumulated levels in several tissues such as liver and lung were very low in high-dose treated group (Fig. 3). As shown in Fig. 2, histopathological changes were not observed in liver, lung and kidney in high-dose treated group and this may be the low accumulated CNP in liver, lung and kidney. On day 1, the level of CNP in lung was significantly elevated (about 35.0 μg/g tissue) but the level was decreased to about (4.5 μg/g tissue) on day 14 when histopathology was performed. It may be possible that pro-inflammatory signs in lung may disappear on day 14 after treatment.

At this moment, we do no have any information on the *in vivo* toxicity of CNP when animals were treated by inhalation, intravenous or intraperitoneal injection. When we treated mice with CNP by intratracheal instillation at the dose of 400 mg/kg, dead animals were not found but serious ROS generation and pro-inflammatory responses were observed (unpublished data). By these results, it seemed that the toxic mechanisms of CNP between *in vivo* and *in vitro* study may be different or the bioavailability may be the critical factor in *in vivo* study of CNP.

## ACKNOWLEDGEMENT

This work was supported by Ministry of Environment as the Eco-technopia 21 project.
